# COVID-19-associated Large Vessel Stroke in a 28-year-old Patient: NETs and Platelets Possible Key Players in Acute Thrombus Formation

**DOI:** 10.1007/s00062-020-00992-1

**Published:** 2021-01-29

**Authors:** Tobias Boeckh-Behrens, Daniel Golkowski, Benno Ikenberg, Jürgen Schlegel, Ulrike Protzer, Christian Schulz, Julia Novotny, Kornelia Kreiser, Claus Zimmer, Bernhard Hemmer, Silke Wunderlich

**Affiliations:** 1 Department of Neuroradiology, School of Medicine, University Hospital rechts der Isar of the Technical University Munich, Ismaninger Str. 22, Munich, 81675 Germany; 2 Department of Neurology, School of Medicine, University Hospital rechts der Isar of the Technical University Munich, Munich, Germany; 3 Institute of Pathology, School of Medicine, University Hospital rechts der Isar of the Technical University Munich, Munich, Germany; 4 Institute of Virology, School of Medicine, University Hospital rechts der Isar of the Technical University Munich, Munich, Germany; 5 Medizinische Klinik and Poliklinik I, LMU Klinikum, Ludwig-Maximilians-Universität, Marchioninistraße 15, 81377 Munich, Germany; 6 Walter-Brendel-Zentrum für Experimentelle Medizin, Ludwig-Maximilians-Universität, Marchioninistraße 27, 81377 Munich, Germany; 7 Munich Cluster for Systems Neurology (SyNergy), Munich, Germany

COVID-19 (corona virus disease-19) is associated with acute stroke in up to 5% of all cases [1] and was initially reported in patients with typical risk factors; however, recent reports also described large vessel occlusions (LVOs) in younger patients with no pre-existing conditions and only mildly affected by COVID-19 [2, 3].

We report the case of a 28-year-old female patient with COVID-19-associated large vessel stroke and the results of the tissue analyses of the extracted clot. Although COVID-19-associated diffuse thrombosis of the microvascular bed has been reported [4], we believe this may be the first reported detailed analysis of extracted clot tissue of a COVID-19-associated LVO beyond mere clinical observations.

## Case

A 28-year-old woman with PCR-proven SARS-CoV‑2 (severe acute respiratory syndrome coronavirus type 2) pneumonia was admitted to our hospital with acute symptoms suggestive of a middle cerebral artery (MCA) occlusion. She had developed SARS-CoV‑2 associated pneumonia 10 days prior to admission and was treated with paracetamol, pantoprazol, metamizol and levofloxacin. On the day of admission, she contacted her general practitioner with progressive dyspnea. In the ambulance heading for a nearby hospital she developed a left-sided hemiparesis and aphasia. There were no other pre-existing conditions or cardiovascular risk factors. Her medical history was positive for bronchial asthma but she did not require medication.

**Fig. 1:**
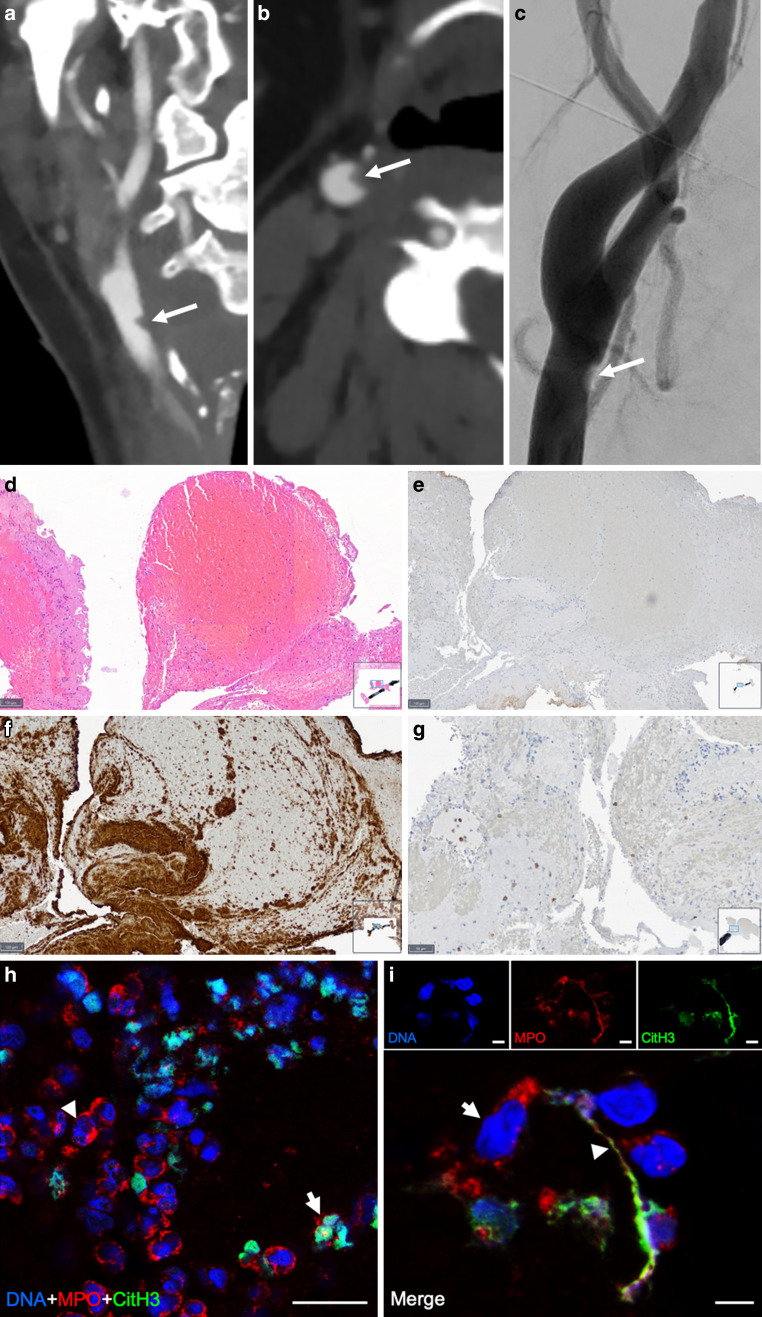
**a** Coronal and **b** axial view of the right common carotid artery (CCA) just before the bifurcation. *Arrow* small, wall-adherent thrombus. **c** Corresponding digital subtraction angiography (DSA) image of the semilunar contrast defect representing the wall-adherent thrombus material (*arrow*). **d**–**i** Histological analyses of the extracted thrombus. **d** Hematoxylin and eosin (HE) staining of platelets with large areas of fresh erythrocyte-rich areas (*red*), considerable numbers of nucleated immune cells (*blue*) and partially interspersed, partially aggregated areas of platelets (*pink*). Bar 100 µm. **e** Fibrinogen staining of the thrombus showing nearly no fibrin inside the thrombus. Bar 150 µm. **f** CD31 staining showing almost all pink areas in the HE staining as positive, suggestive of nearly exclusive thrombocyte aggregations with only minimal fibrin proportions. Bar 100 µm. **g** CD45 staining polymorphonuclear cells (PMNs) shows only a minority of intrathrombus immune cells being lymphocytes suggesting a predominance of PMNs. Bar 50 µm. **h** Overview image of citrullinated histone H3 positive (CitH3+) neutrophils (Myeloperoxidase [MPO] *red*; CitH3+ *green*). *Arrowhead* on MPO+ neutrophil; *arrow* on CitH3/MPO+ neutrophil. Nuclei are counterstained with Hoechst (*blue*). Bar 20 µm. **i** Detailed view of immunohistochemical staining of CitH3+ neutrophil extracellular trap (MPO, *red*; citrullinated histone H3, *green*). Nuclei are counterstained with Hoechst (*blue*). Extracellular DNA originates from MPO+ neutrophil. *Arrow*, nucleus; *arrowhead*, neutrophil extracellular traps (NET) fiber. Bars, 5 µm

In the external hospital laboratory tests showed lymphopenia (20% decrease, no exact value available), elevated D‑dimers (17.81 mg/l), elevated thrombocyte count (615 * 10^3^/µl), elevated liver enzymes (alanine aminotransferase [ALT] 38 U/l, aspartate aminotransferase [AST] 53 U/l), lactate dehydrogenase (LDH) (497 U/l) and C-reactive protein (CRP) (23.39 mg/dl) suggesting bacterial superinfection. Cranial computed tomography (CT) with CT angiography showed no signs of brain infarction but an occlusion of the right middle cerebral artery (MCA) and a wall-adherent thrombus formation at the distal common carotid artery (CCA) as probable stroke cause (Fig. 1a–c). The patient was then transferred to our department, where she presented with an NIH stroke scale of 15 points. She received 67.5 mg of rtPA (recombinant tissue plasminogen activator) 3h after symptom onset. Mechanical thrombectomy in the M1 segment of the MCA using a combined approach with stentretriever, distal aspiration and proximal flow control using a ballon guide catheter (BGC) was successful 3.5 h after symptom onset (see supplemental Fig. 1) and the clot material could be retained for further histopathological analysis. The slight proximal thrombus residues at the distal CCA (see Fig. 1c) were left untouched. Within 6 days the patient improved to an NIHSS of 3 points and was discharged to rehabilitation with no requirement of ventilation at any time and nearly no persisting COVID-19-related symptoms. Possible concurrent stroke causes were excluded as described in the supplement (supplemental Fig. 2).

The PCR analysis detected no virus-specific RNA inside the thrombus material using *N*-gene specific primers. Morphological analysis using standard hematoxylin-eosin (HE) and immunostainings are shown and described in Fig. 1d–g. In brief, the clot had an hyperacute appearance with large amounts of platelets and erythrocytes and unusually minimal amounts of fibrin/fibrinogen.

Immunofluorescence analysis showed a large number of neutrophils and neutrophil extracellular traps (NETs), a key factor of early thrombogenesis and link between excessive activation of immunological processes and thrombus formation known as immuno-thrombosis (Fig. 1h, i). Of the thrombus neutrophils 25% were primed to produce NETs as indicated by citrullinated histone H3 (citH3) staining.

## Discussion

Vascular complications during the course of CoV‑2 infections are possibly due to interconnected pathophysiological processes: a direct virus-associated endotheliitis [4] resulting in microcirculatory dysfunction, thrombosis and a local thromboinflammatory state, leading to a global severe hypercoagulability [5].

However, it is still not clear which vascular structures and which parts of the blood coagulation system and the immunothrombotic link are predominantly involved. Previous reports mainly reported endothelial damage in microvascular compartments [4]. Also, a direct infection or activation of platelets by the virus via the ACE2 (angiotensin-converting enzyme 2) receptor with procoagulatory effects is discussed [6]. We here provide evidence for affection of macrovascular vessel walls, possibly representing endothelial damage leading to large vessel strokes.

The considerable NET amount, although previously described [7], in conjunction with the low fibrinogen/fibrin amount and the predominance of platelets is unusual and hints at an endothelial damage-driven, neutrophil-platelet interaction as key process of thrombogenesis in this specific situation. The NET activity in COVID-19 patients seems also to be associated to or even trigger other critical and prognosis defining effects in the course of the disease, e.g. necessity of ventilation, increased mucus viscosity, ARDS-like changes and others [8, 9]. This notion is further supported by our findings.

The main findings and possible conclusions of the presented case can be summarized and discussed as follows:

1. Although one case description cannot provide any absolute proof, the presented case suggests, that SARS-CoV‑2 related LVOs might occur also in patients without any cardiovascular risk factors and also in not severely COVID-19 affected patients.
2. In the absence of other evident etiologies, the underlying stroke cause in our case was a local arteriopathy in a high-flow large artery (CCA) without any visible predamage that is compatible in principle with global endothelial inflammatory damage also in large vessels, in line with previous reports [3, 10]. As limitation, we have to take the (at least remote) possibility into consideration, that the hypercoagulable state was related to a paradoxical effect of the above described medication [11].
3. Clot analyses direct towards a hyperacute and inflammation-linked thrombus formation, probably predominantly based on platelet-neutrophil interactions including NET formation as one underlying key process.
4. No evidence for a direct virus-mediated procoagulatory effect within the thrombus could be obtained.

Taken together, the case we report here and the molecular tissue analysis further underline the importance of further studies evaluating the most appropriate and target specific anticlotting strategies (e.g. antiplatelet agents) with a special focus on platelet function, platelet-neutrophil interaction and NETosis in the course of COVID-19.

## Supplementary Information

The Supplementary Information contains additional image material showing the digital subtraction angiography of the interventional procedure and the post intervention magnetic resonance imaging showing the infarction. Additionally, all diagnostic procedures to exclude concurrent stroke causes are described.
