# Supplementary material for: COVID-19-associated Large Vessel Stroke in a 28-year-old Patient: NETs and Platelets Possible Key Players in Acute Thrombus Formation
Source: Clin Neuroradiol. 2021 Jan 29;31(2):511–4. doi: 10.1007/s00062-020-00992-1 (PMC7844783; doi:10.1007/s00062-020-00992-1)
Supplement: Supplementary file 1 — The Supplementary Information contains additional image material showing the digital subtraction angiography of the interventional procedure and the post intervention magnetic resonance imaging showing the infarction. Additionally, all diagnostic procedures to exclude concurrent stroke causes are described. [file 62_2020_992_MOESM1_ESM.docx]

Supplementary Material


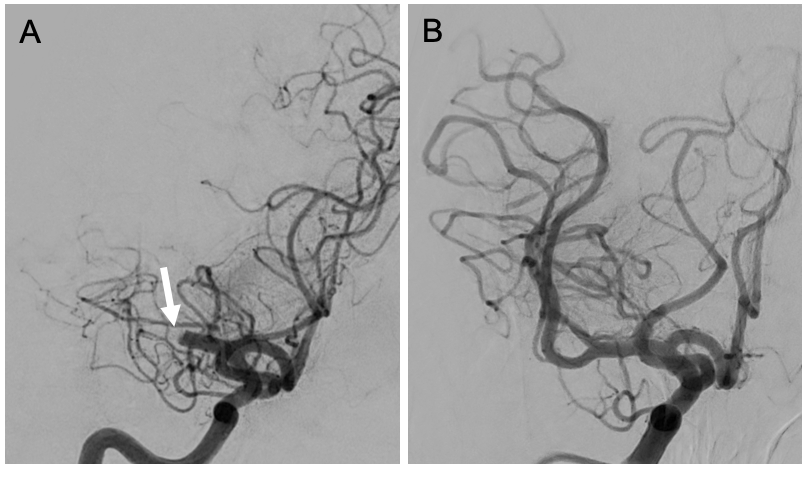


Supplemental Figure 1. (A) DSA: Sharp truncation of the M1 segment of the right middle cerebral artery (MCA) in location of the occlusion (arrow)

(B) Normal anatomical situation after mechanical extraction of the embolus. Note the regular shape of the M1 segment in location of the previous occlusion without signs of a local arteriopathy.

Possible concurrent stroke causes were excluded as follows:

A cranial MRI 24 hours after symptom onset demonstrated a partial infarction of the medial right MCA territory (Supplemental Figure A and B) and showed no sign of cerebral vascular disease or pathology outside the right MCA territory. Ultrasonography of the heart remained unremarkable, in particular no patent foramen ovale was detected. Electrocardiographic monitoring for 72 hours showed regular sinus rhythm. A lumbar puncture had normal cell count, glucose, lactate (1.6 mmol/l) and protein fractions. The cerebrospinal fluid PCR for SARS-CoV-2 was negative and there was no intrathecal synthesis of IgG, IgA or IgM. Antibodies against cardiolipin, beta_2_ glycoprotein, double-stranded DNA and a screening for anti-nuclear antibodies were normal.

Supplemental Figure 2. Magnetic resonance imaging 24 hours after symptom onset.

A. A fluid attenuated inverse recovery (FLAIR) sequence showing hyperintense signal in the basal ganglia, mainly the putamen and pallidum, and the insular cortex. B. A diffusion weighted image (DWI) showing hyperintense signal encompassing parts of the right MCA territory.
